# Novel Genetic Variants of the *CHD2* Gene in *cis* Position in a Patient with Epileptic Encephalopathy: A Case Report and In Silico Analysis of the Impact of the Variants on the Protein

**DOI:** 10.3390/ijms27209120

**Published:** 2026-10-14

**Authors:** Andrew S. Zuev, Elizaveta A. Fonova, Daria S. Kuznetsova, Vadim A. Stepanov, Nikolay A. Skryabin

**Affiliations:** 1 Research Institute of Medical Genetics, Tomsk National Research Medical Center, Tomsk 634009, Russia; andrew.zuev@medgenetics.ru (A.S.Z.); daria.kuznetsova1701@gmail.com (D.S.K.); vadim.stepanov@medgenetics.ru (V.A.S.); nikolay.skryabin@medgenetics.ru (N.A.S.); 2 Department of Medical Genetics, Siberian State Medical University, Tomsk 634050, Russia

**Keywords:** *CHD2*, c.1634T>G, c.3810G>C, epilepsy, eyelid myoclonia with absences, Jeavons syndrome, nanopore sequencing, variant phasing, protein stability

## Abstract

*CHD2*-related neurodevelopmental disorders are increasingly recognized as a distinct group of epileptic encephalopathies with heterogeneous clinical manifestations. Here, we report a 12-year-old female patient with pharmacoresistant epilepsy, mild cognitive impairment (IQ = 56), and extraneurological features including grade II kyphoscoliosis and planovalgus foot deformity. Video-EEG monitoring revealed a phenotype consistent with Jeavons syndrome, characterized by daily eyelid myoclonia with absences and marked photosensitivity with an eye closure sensitivity (ECS) phenomenon. Molecular genetic testing via whole-exome and Sanger sequencing identified two novel variants in the *CHD2* gene (NM_001271.4): c.1634T>G (p.Leu545Arg) and c.3810G>C (p.Leu1270Phe). Long-read nanopore sequencing confirmed their de novo status and *cis*-positioning on the paternal allele. Structural bioinformatic analysis using AlphaFold3, FoldX/mCSM (v5.0 package), UCSF ChimeraX (v1.10.1), and PyMOL (v3.1.8) demonstrated that both variants individually destabilize the CHD2 protein, with a cumulative destabilizing effect when combined: p.Leu545Arg affects the ATP-binding domain, whereas p.Leu1270Phe disrupts hydrophobic packing within the C-terminal DNA-binding domain. These findings suggest accelerated protein degradation as a potential disease mechanism. Our data support the pathogenicity of these heterozygous *cis*-variants and implicate *CHD2* haploinsufficiency as the leading etiopathogenic mechanism, though distinct domain-specific effects of each substitution may also contribute to the complex molecular synergy observed in this case.

## 1. Introduction

Chromatin remodeling represents a fundamental epigenetic mechanism that governs the dynamic regulation of transcriptional programs essential for neurogenesis, synaptic plasticity, and maintenance of neuronal identity. Among the key players in this process is the chromodomain-helicase-DNA-binding (CHD) family of proteins, which utilize ATP-dependent nucleosome remodeling to modulate gene expression [1,2]. The *CHD2* gene (OMIM: 602119) encodes a member of this family that is highly expressed in the developing and mature brain, playing a critical role in neuronal differentiation and cortical lamination [3,4]. Pathogenic variants in *CHD2* have been increasingly associated with a broad spectrum of severe neurodevelopmental and epileptic disorders, including developmental and epileptic encephalopathy type 94 (DEE94, OMIM: 615369), intellectual disability, autism spectrum disorder, and various epilepsy syndromes [5,6]. The phenotypic spectrum ranges from mild learning difficulties to profound developmental delay with early-onset, pharmacoresistant seizures, often exacerbated by fever or photic stimulation [7]. Despite growing awareness, the molecular mechanisms underlying *CHD2*-related pathology remain incompletely understood, particularly regarding the functional consequences of missense variants located in functionally distinct domains of the protein.

In this study, we describe a clinically well-characterized case of *CHD2*-associated pathology in a 12-year-old girl presenting with Jeavons syndrome, a distinctive form of generalized epilepsy characterized by eyelid myoclonia with absences and photosensitivity, in combination with mild cognitive deficit and skeletal abnormalities. We employed a multi-layered approach combining comprehensive clinical phenotyping, video-EEG monitoring, neuroimaging, whole-exome and Sanger sequencing, long-read nanopore-based phasing, and advanced structural bioinformatic modeling. Our aim was to assess the pathogenetic significance of two novel heterozygous *cis*-variants and to explore their potential additive effects on CHD2 protein stability and domain function.

## 2. Patient Report

The patient is a 12-year-old girl of Caucasian ethnicity. She was born from a first pregnancy that proceeded without complications. According to parental reports, psychomotor development up to 3 years of age was age-appropriate. At 2.5 years of age, behavioral changes were first noted, including irritability, aggressiveness, and sleep disturbances. At 3 years of age, epileptic seizures manifested as eyelid absences (eye squeezing, gaze freezing) lasting 3–6 s, with a frequency exceeding 50 episodes per day. Subsequently, regression of motor skills was observed (development of an awkward gait). From 4 years of age, sodium valproate (Depakine) therapy was initiated. During treatment, seizure frequency decreased approximately twofold, and seizure duration was reduced to 2–3 s. After 2 months, eyelid myoclonia appeared, leading to the additional prescription of lamotrigine (Lamictal). At 9 years of age, valproate was discontinued and replaced with ethosuximide (Petnidan).

The medical history included musculoskeletal abnormalities, such as valgus deformity of the lower extremities and planovalgus foot deformity, noted since 2 years of age. From 6 years of age, posture impairment (grade II kyphoscoliosis) has been observed. Physical examination revealed a hyperpigmented leaf-shaped spot (2–3 cm) on the back and single neoplasms up to 1 cm with moderate hypertrophy and altered relief on the lumbar region. Minor dysmorphic features were identified, specifically an altered auricular helix shape and anteriorly rotated earlobes. Visual impairment (mild hypermetropia of both eyes) was also noted.

Neuropsychological assessment using the Wechsler Intelligence Scale for Children (WISC) yielded the following scores: verbal intelligence index—66, nonverbal—53, and full-scale IQ was 56. This result corresponds to mild intellectual disability according to ICD-10 criteria (F70). In the context of modern classifications (DSM-5), the condition qualifies as mild intellectual developmental disorder (IQ level 55–70, considering clinically observed adaptive behavior impairments).

Brain magnetic resonance imaging (MRI) and multislice computed tomography (MSCT) performed at 7 years of age revealed no significant structural pathology or focal brain parenchymal changes. Prolonged video-EEG monitoring performed at 10 years of age demonstrated pronounced ictal epileptiform activity during both sleep and wakefulness. This activity was represented by high-amplitude diffuse symmetric and asymmetric discharges of single and grouped “spike–polyspike–wave” complexes with regular bilateral predominance in the frontal polar regions. In one-third of cases, these discharges were accompanied by motor equivalents in the form of brief eyelid myoclonia or eye squeezing, as documented by simultaneous video recording. Trigger dependence of ictal discharge occurrence with light cessation or eye closure by the patient ECS was established. Interictal epileptiform activity was characterized by a low representation index throughout the recording, with attenuation during active wakefulness. This activity was not accompanied by visible clinical manifestations and was represented by three independent foci with frequent diffuse irradiation: single regional spikes and “spike–wave” complexes in the left posterior temporal region (leads O1, P3, T5); single regional spikes and “spike–wave” complexes in the right anterior-mid temporal region (leads F8, T4); single regional spikes, polyspikes, and “spike–wave” complexes in the right posterior temporo-parietal region (leads P4, T6). During provocative testing, ictal activity and motor paroxysms were provoked by photic stimulation across the entire range of presented frequencies, and during hyperventilation, a substantial increase in the ictal activity index with manifestation of the ECS phenomenon was recorded. Retrospective comparison with EEG data at 9 years of age revealed marked worsening, manifesting as a moderate increase in amplitude and index of both diffuse ictal and regional interictal discharges during sleep and wakefulness.

To establish the genetic etiology of the disease, whole-exome sequencing of DNA was performed at 9 years of age. High-throughput sequencing of genomic DNA regions corresponding to exons and splice sites was performed on the HiSeq 1500 platform (Illumina, San Diego, CA, USA) using the SureSelect Human All Exon V7 target enrichment panel (Agilent Technologies, Santa Clara, CA, USA) at the Genoanalytika LLC laboratory (Moscow). Bioinformatic analysis identified two previously undescribed nucleotide sequence variants in the *CHD2* gene. The identified variants were interpreted as likely pathogenic and potentially relevant to the patient’s phenotype, pending additional confirmatory data. Both variants were confirmed as de novo in the patient following trio Sanger sequencing at the same laboratory. Based on the correlation of genotyping results with the clinical presentation, the geneticist concluded that the proband’s phenotypic manifestations correspond to the symptom complex of *CHD2*-associated epileptic encephalopathy.

Currently, the patient is receiving combined antiepileptic therapy including lamotrigine at a daily dose of 300 mg and ethosuximide at a daily dose of 1000 mg. On this treatment regimen, a partial therapeutic response is observed: seizures persist daily, with a frequency of 5 to 15 paroxysms per day, each episode lasting 2–4 s. Seizure semiology is represented by isolated eyelid myoclonia, as well as eyelid myoclonia combined with absences, occasionally accompanied by tonic upward deviation of the eyes with partially open palpebral fissures. Clinical analysis demonstrated a correlation between paroxysm manifestation and sleep deprivation, physical fatigue, psycho-emotional stress, and light-related triggers (natural or artificial lighting).

Repeated analysis of the clinical significance of the identified *CHD2* variants led to the classification of the c.1634T>G variant as likely pathogenic (ACMG criteria: PS2, PM2, PP2, PP3) and the c.3810G>C variant as pathogenic (ACMG criteria: PS2, PM1, PM2, PP2, PP3). Both variants were confirmed to be de novo with confirmed maternity and paternity (PS2) and were absent from population databases including gnomAD, 1000 Genomes, and ExAC (PM2). Because both are missense changes in a gene with a low rate of benign missense variation, in which missense variants are a common mechanism of disease (PP2), and because multiple lines of computational evidence support a deleterious effect (PP3), the c.1634T>G variant was classified as likely pathogenic. The c.3810G>C variant satisfies identical criteria (PS2, PM2, PP2, and PP3) and meets the PM1 criterion, as it resides within a critical mutational hotspot lacking known benign variation. Specifically, this 151-bp region in exon 30 (chr15:92997252–92997403) encompasses six reported pathogenic or likely pathogenic variants, with an absence of benign missense alterations in ClinVar. Taken together, these findings justify its classification as pathogenic.

## 3. Results

### 3.1. Long-Read Sequencing

The number of reads covering the target loci ranged from 4888 to 5785 (region c.1634) and from 4037 to 6372 (region c.3810) across the three family members. At variant positions, depth reached 2118–2731× (c.1634T>G, chr15:92953488) and 3018–4644× (c.3810G>C, chr15:92997328). Read quality metrics were stable across all samples: mean Phred quality score ranged from 15.72 to 16.37, Q20 read proportion ranged from 79.8% to 82.3%, and Q30 read proportion ranged from 57.1% to 63.3%. Read length N50 was 8049–8050 bp (c.1634T>G) and 6736–6737 bp (c.3810G>C). All identified variants had PASS status in Clair3 v2.0.2 output. Allele ratios were approximately 46–47% for both variants. The results obtained were additionally verified on unprocessed BAM files to exclude filtering artifacts. For unambiguous determination of parental haplotype origin, informative SNP markers were used in which one parent was homozygous for the alternative allele (1/1), enabling definitive chromosome assignment. In both investigated variant regions, such markers were variants with a homozygous 1/1 genotype in the mother (Table 1).

Both variants were absent in parents (0/0), confirming their de novo status in the proband. Allele phasing in the trio established that both de novo variants (c.1634T>G at position 92953488 and c.3810G>C at position 92997328) are located in *cis* configuration on the paternal copy of chromosome 15. Parental origin was confirmed by independent SNP markers with maternal homozygosity (1/1).

### 3.2. In Silico Analysis of CHD2

**Figure 1:**
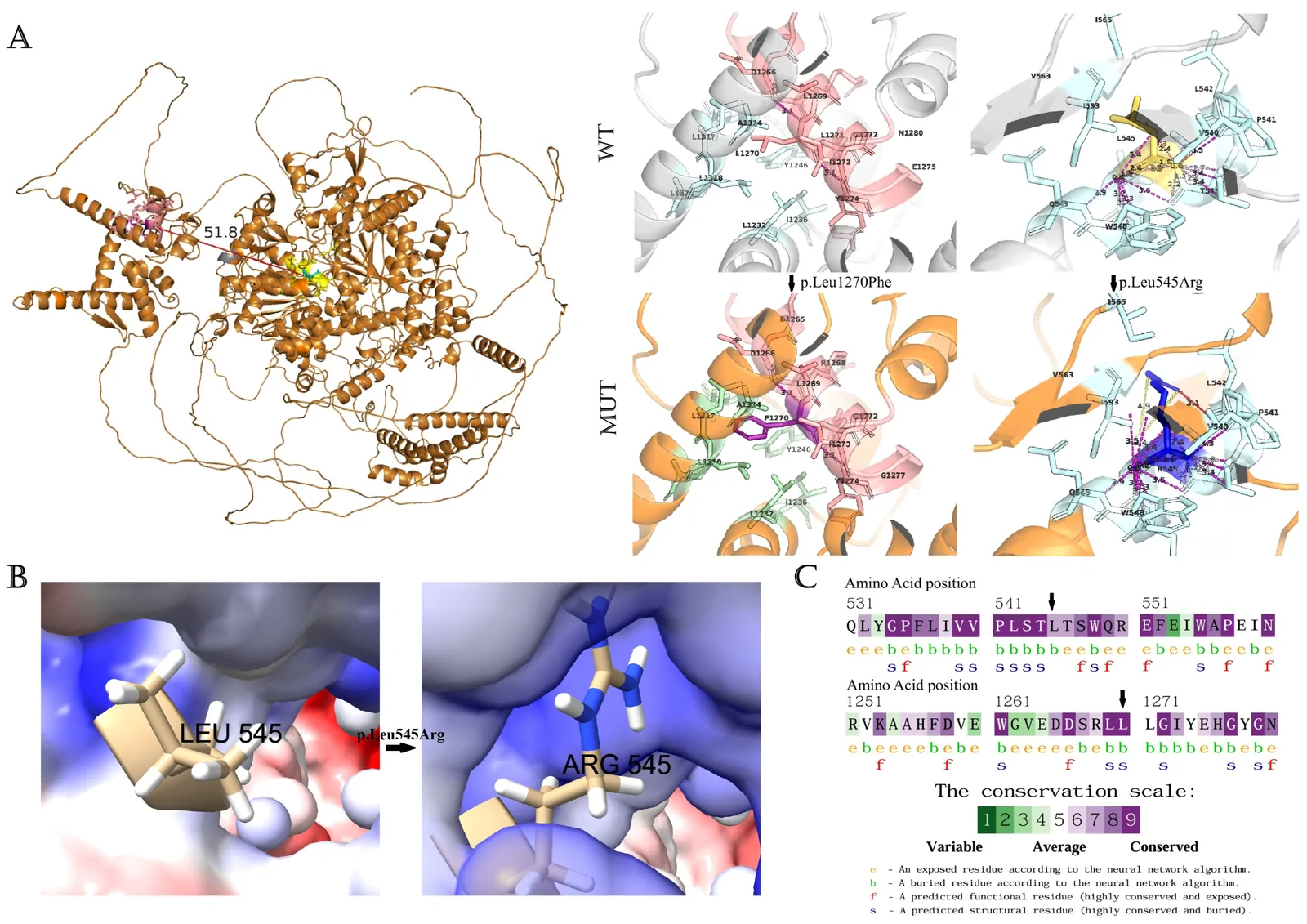
Structural and evolutionary consequences of the cis haplotype p.Leu545Arg + p.Leu1270Phe in the CHD2 protein. (**A**) Overall protein architecture and microenvironment of variant positions. The full-length CHD2 model (AF-O14647-F1-model_v6, FoldX-optimized) is shown as an overlay of the unchanged protein (gray) on the double mutant protein (orange). The red dashed line indicates the distance between Cα atoms of amino acid positions 545 and 1270. Position 545 (helicase ATP-binding domain) is highlighted in yellow/blue; position 1270 (C-terminal DNA-binding domain) in pink/purple. Microenvironments at the variant positions are shown as images in the unchanged protein (**upper row**) and with variants (**lower row**). Pink indicates the α-helix 1260–1280 and polar/charged neighbors of position 1270; purple indicates the p.Leu1270Phe substitution; green shows the hydrophobic cluster of the DNA-binding domain into which residue 1270 is embedded. At position 545, blue indicates the hydrophobic environment, yellow indicates Leu545 (WT), and blue indicates Arg545 (mutant). In WT, only van der Waals interactions (purple dashed line) are visible around Leu545 in the hydrophobic patch; in the mutant, the guanidinium group of Arg545 forms new hydrogen bonds (red dashed line, 3.1 Å with O Leu542), while positively charged Lys847 remains nearby (yellow dashed line, 4.9 Å), a source of electrostatic repulsion “+/+”. (**B**) Electrostatic surface in the region of position 545. Left: Leu545 is located in a zone without pronounced charge. Right: Arg545 forms a local positive patch absent in WT. Blue indicates positive potential, red—negative, and white—neutral. (**C**) Evolutionary conservation of variant positions (ConSurf). Sequence fragments 531–560 and 1251–1280 are colored according to the conservation scale 1–9 (green—variable, purple—conserved). Black arrows indicate variant positions.

Analysis of CHD2 protein domain architecture using UniProt, InterPro, and structural–functional studies [6] revealed that the variants are localized in two distinct functional domains: the helicase ATP-binding domain (p.Leu545Arg) and the C-terminal DNA-binding domain (p.Leu1270Phe). To assess the structural context of the variants, the predicted AlphaFold model of the full-length protein (AF-O14647-F1, v6) was used. Model quality at variant positions was confirmed by pLDDT values of 87.44 for position 545 and 88.62 for position 1270. Structural alignment of wild-type (WT) and double *cis*-mutant (p.Leu545Arg + p.Leu1270Phe) models demonstrated preservation of global protein folding (RMSD = 0.000 Å over 12,314 atoms). The distance between Cα atoms of amino acid positions 545 and 1270 was 51.8 Å, indicating the absence of direct spatial contacts. These structural data indicate the absence of global conformational rearrangements, suggesting that the effects of variants may be local and structurally independent (Figure 1A).

For quantitative assessment of the effects of amino acid substitution on protein stability, changes in folding free energy (ΔΔG) were calculated using FoldX (Table 2).

The epistasis value of +0.46 kcal/mol indicates a virtually additive nature of the effects of two mutations, consistent with their spatial separation and the absence of direct interactions. To verify FoldX results, an independent assessment of the effects of each missense substitution on protein stability was performed using four additional algorithms, all of which unanimously predicted thermodynamic destabilization of the CHD2 mutants (Table 3).

Analysis of the microenvironment of residue Leu545 revealed partial solvent accessibility (SASA = 109.6 Å^2^) and its location at the boundary of a hydrophobic patch. The amino acid environment of position 545 (within a 5 Å radius) revealed a pronounced hydrophobic cluster (VAL540, PRO541, LEU542, VAL563, ILE565, ILE593, TRP548), the presence of positively charged amino acids (LYS847, electrostatic interaction with ARG545), hydrogen bonds (Arg545 side chain with NH1-O Leu542), and the absence of salt bridges and steric clashes. On the electrostatic surface of the double *cis* mutant in the region of position 545, a local positive (blue) patch appears that is more pronounced than in the WT model (Figure 1B).

Analysis of the microenvironment (within a 5 Å radius) of position 1270 revealed its location within a hydrophobic cluster (LEU1232, ILE1236, TYR1246, LEU1248, LEU1269, LEU1271, LEU1314, LEU1317, LEU1318, LEU1321) in an α-helix. Substitution of leucine with the bulkier phenylalanine preserves the backbone hydrogen bonds of the α-helix and does not cause steric clashes; however, it disrupts the tight hydrophobic packing of the cluster, manifested as increased SASA (129.8 Å^2^ for Phe1270 vs. 114.3 Å^2^ for Leu1270) and local loosening of the structure.

Evolutionary conservation analysis using the ConSurf server revealed that both positions are highly conserved (Table 4, Figure 1C).

## 4. Discussion

The association of various *CHD2* gene variants with neurodevelopmental and functional disorders has been demonstrated in numerous studies [7,8,9,10]. These disorders are underpinned by dysfunction of the encoded protein directly within neuronal system cells. The CHD2 protein possesses a complex multidomain architecture that determines its molecular activity. The presence of two tandem chromodomains at the N-terminus ensures recognition and specific binding of modified histone tails, while the central SNF2-like ATPase/helicase domain performs ATP hydrolysis, providing energy for chromatin structure alteration. The C-terminal DNA-binding domain is responsible for direct binding to double-stranded DNA [6]. The coordinated functioning of these structural units ensures nucleosome assembly and mobilization, allowing dynamic reorganization of the cellular transcriptional landscape, thereby mediating tight regulation of neurogenesis and neuronal stem cell proliferation [11]. It has been experimentally demonstrated that *CHD2* haploinsufficiency induces pronounced downregulation of key cell cycle genes alongside premature activation of transcriptional programs specific to mature neurons. This imbalance leads to premature differentiation of cortical interneurons [12]. In heterozygous *CHD2* mouse models, it was shown that such changes not only suppress neuronal progenitor proliferation but also severely disrupt synaptic transmission, causing multidirectional shifts in the balance of excitatory and inhibitory neuronal systems [13]. The exceptional importance of *CHD2* for human neurodevelopment is underscored by differences in its expression across all stages of ontogenesis, as well as by the fact that pathogenic variants in this gene manifest predominantly with nervous system phenotypes [4].

According to OMIM (#615369) and clinical studies, the spectrum of neurodevelopmental disorders associated with *CHD2* gene variants is characterized by pronounced heterogeneity. The leading clinical pattern in developmental encephalopathy and epileptic encephalopathy type 94 (DEE94) is a combination of pharmacoresistant epileptic seizures of various types, psychomotor developmental delay and regression, autism spectrum disorders, and intellectual disability of varying severity. Several authors also emphasize the pathognomonic high frequency of photosensitivity and febrile seizure provocation in this patient group. The clinical phenotype of our patient demonstrates a high degree of specificity and is fully consistent with the classical framework of *CHD2*-associated pathology, confirmed by the presence of eyelid myoclonia with absences, pronounced photosensitivity (Jeavons syndrome) [14], and mild cognitive deficit (Table 5).

Of particular interest in the presented case are the extraneurological manifestations involving the musculoskeletal system (valgus deformity of the lower extremities and grade II kyphoscoliosis). It can be hypothesized that the observed musculoskeletal abnormalities in the proband are etiologically linked to the identified *CHD2* variants. This hypothesis is consistent with the work of Kulkarni et al., who, based on an analysis of mouse models and clinical observations, substantiated the role of *CHD2* haploinsufficiency in the development of multisystem anomalies in humans, including the formation of severe scoliosis [15]. From a molecular-biological perspective, the systemic pleiotropic effect of the variants identified in our patient may be attributable to the cumulative disruption of tertiary structure and protein stability. Reduced CHD2 stability can impair common epigenetic differentiation programs not only in neuronal pathways but also in mesenchymal cell lineages, leading to connective tissue dysplasia and skeletal deformities.

The evolutionary conservation data for both investigated positions obtained during bioinformatic analysis (GERP/PhastCons score of 8–9 points) indicate pronounced evolutionary pressure against amino acid substitutions at these loci. These findings confirm their critical functional and structural significance, particularly given the extremely low tolerance of *CHD2* to missense variants at the population level (Z-score = 7.47 according to the gnomAD v4.1.1 database). The variants identified on the paternal allele are localized in two distinct functional domains of the CHD2 protein, exerting an additive effect on its spatial configuration. The appearance of an uncompensated positive charge upon substitution of nonpolar leucine with hydrophilic arginine at position 545 (p.Leu545Arg) within the hydrophobic core of the domain induces extreme thermodynamic destabilization of the structure (ΔΔG = +9.63 kcal/mol). This alteration most likely disrupts ATP coordination, binding, and/or subsequent hydrolysis, thereby impairing the key function of the helicase ATP-binding domain. The substitution of leucine with the bulkier phenylalanine in the structural core of the C-terminal DNA-binding domain (p.Leu1270Phe, ΔΔG = +5.02 kcal/mol), although preserving backbone hydrogen bonds of the α-helix without generating direct steric clashes, severely disrupts the tight hydrophobic packing of the local cluster. This is clearly manifested by an increase in solvent-accessible surface area (SASA +15.5 Å^2^) and may critically reduce both the stability of the DNA-binding module itself and its affinity for nucleosomal DNA. The cumulative effect of the two variants on a single polypeptide chain (ΔΔG_double = +15.11 kcal/mol) is interpreted as critical destabilization of the macromolecular tertiary structure. Such a degree of conformational disruption is hypothesized to trigger accelerated degradation of the mutant protein by the intracellular ubiquitin proteasome system. However, it should be emphasized that this degradation mechanism remains purely theoretical and *lacks* in vitro or in vivo experimental validation. Given these structural alterations, as well as the presence of an intact gene copy on the maternal homolog, the primary molecular mechanism of disease development in the described clinical case should be regarded as functional haploinsufficiency of the *CHD2* gene. Nevertheless, alternative molecular pathogenic pathways—beyond the independent functional impairment induced by each variant—cannot be entirely excluded.

## 5. Materials and Methods

The research was conducted in accordance with the ethical standards of the World Medical Association’s Declaration of Helsinki. Informed consent was obtained from all family members prior to their participation. The study protocol was approved by the Biomedical Ethics Committee of the Research Institute of Medical Genetics at the Tomsk National Research Medical Center (Approval Code: 10, 15 February 2021). The family applied to the Research Institute of Medical Genetics, Tomsk National Research Medical Center (NRMC), for reanalysis of whole-exome sequencing (WES) results obtained from an external laboratory and for additional molecular genetic diagnostics. Additional studies were conducted at the Core Medical Genomics Facility of the Tomsk National Research Medical Center (Tomsk NRMC) of the Russian Academy of Sciences.

The collection and analysis of clinical and anamnestic data were carried out retrospectively based on a detailed review of the patient’s medical records and investigation results. Clinical examination was performed by a multidisciplinary team of specialists, including a neurologist, epileptologist, geneticist, psychologist, dermatologist, and orthopedist. The recorded information covered family history, early developmental features, somatic status, as well as the nature of pharmacotherapy and its efficacy assessment. Epileptic seizure types were classified according to the criteria of the International League Against Epilepsy (ILAE).

The instrumental diagnostic data package included results from prolonged video-EEG monitoring (including wakefulness and sleep recordings, as well as rhythmic photic stimulation testing) and high-resolution brain magnetic resonance imaging (MRI). The EEG data obtained in this study are available upon request. Molecular genetic testing by whole-exome sequencing (WES) with subsequent validation by Sanger sequencing and bioinformatic data analysis was performed at the Genoanalytika Laboratory (Moscow, Russia).

### 5.1. Long-Read DNA Sequencing In Silico Analysis

For variant phasing, long-read DNA sequencing was performed on the nanopore-based G-seq500 platform (Geneus Technologies, Chengdu, China), utilizing SNS (Sequencing by Synthesis on Nanopore) technology with a sequencing throughput of >50 Gb (5 Gb per run). To generate extended templates covering the target loci of the *CHD2* gene, long-range PCR amplification was performed. Specific primers flanking target regions longer than 5 kb were designed for each locus separately (Table 6).

Amplicons were obtained using the BioMaster LR HS-PCR kit (Biolabmix, Novosibirsk, Russia), and their purification was performed using a DNA/RNA extraction kit from reaction mixtures (Biolabmix, Russia). DNA libraries were prepared using library preparation, incubation, and magnetic bead kits (Geneus Technologies Co., Ltd., China) according to the manufacturer’s recommendations, without biological replicates of the samples. Sequencing was performed using the highly integrated MK02 chip (512,000 nanopores). Read preprocessing included adapter trimming (Porechop v0.2.4) and quality- and length-based filtering (chopper v0.13.0, threshold Q ≥ 15, minimum length 1200 bp). Filtered reads were aligned to the human reference genome (GRCh38) in the *CHD2* gene region (chromosome 15) using minimap2 v2.31 in map-ont mode (flag-Y to preserve soft-clipping); BAM file processing was performed using samtools v1.21. Single-nucleotide variant calling and phasing were performed using Clair3 v2.0.2 (ont model) with the integrated WhatsHap module. For the identified de novo variants, parental chromosome assignment was established by matching the proband’s phase block (phase set field) with parental genotypes at the same locus. Additionally, manual variant verification was performed by direct allele counting (samtools mpileup) in both raw and processed parental data to exclude filtering artifacts.

### 5.2. In Silico Analysis

Annotation of the identified missense variants in the *CHD2* gene was performed relative to the reference transcript NM_001271.4 and the corresponding protein sequence (UniProt ID: O14647). The correspondence of nucleotide substitutions (c.1634T>G and c.3810G>C) to amino acid consequences (p.Leu545Arg and p.Leu1270Phe) was verified using the Ensembl Variant Effect Predictor (VEP) and manual alignment of the coding sequence. The three-dimensional model of the full-length CHD2 protein was obtained from the AlphaFold Protein Structure Database (AF-O14647-F1, model v6). The model was loaded in mmCIF format and converted to PDB format using the PyMOL 3.1.8 molecular visualization system (Schrödinger, LLC, New York, NY, USA). Model quality at variant sites was assessed using pLDDT (predicted Local Distance Difference Test) values. Preliminary structure preparation (addition of missing atoms, side-chain optimization, hydrogen addition) was performed using the RepairPDB module of the FoldX v5.0 package (https://foldxsuite.crg.eu/, accessed on 31 August 2026) with default parameters.

Mutant model generation was performed in PyMOL v2.5 using the Mutagenesis Wizard tool, which performs amino acid substitution with automatic selection of the optimal rotamer based on the Dunbrack rotamer library. Four models were constructed: WT—wild-type CHD2 protein (Leu545/Leu1270); Mut1—single mutant p.Leu545Arg; Mut2—single mutant p.Leu1270Phe; Paternal_cis_double—double mutant containing both substitutions in a single polypeptide chain (p.Leu545Arg + p.Leu1270Phe), modeling the protein product of the paternal allele. Single mutants (Mut1, Mut2) were used as technical controls to assess additivity of effects.

Assessment of folding free energy change (ΔΔG) upon amino acid substitutions was performed using FoldX v5.0 (Stability command). For each of the four models (WT, Mut1, Mut2, Double), the RepairPDB procedure was preliminarily applied, followed by total energy calculation (kcal/mol). ΔΔG values were calculated as ΔΔG = E_mutant − E_WT. For quantitative assessment of epistatic interaction between the two substitutions, ΔΔG_expected = ΔΔG_L545R + ΔΔG_L1270F and ΔΔG_epistasis = ΔΔG_double − ΔΔG_expected were calculated. To verify FoldX results, independent assessment of missense substitution effects on protein stability was performed using the mCSM, SDM, DynaMut2, and DUET web servers.

Electrostatic potential visualization was performed in UCSF ChimeraX 1.10.1 using the Coulombic Surface Coloring tool. Hydrogen atoms were preliminarily added using the AddH tool. Intramolecular hydrogen bond (H-bond) search in WT and double mutant models was performed using the Find H-Bonds tool with standard geometric criteria (donor–acceptor distance ≤ 3.5 Å, D-H⋯A angle ≥ 120°) with relaxation of constraints by 0.4 Å and 20°. Salt bridge search was performed in salt bridges only mode. Results were manually verified using ChimeraX log file output.

Functional annotation of CHD2 protein domains and determination of variant localization was performed based on data from the UniProt database, the InterPro domain integration database, and published structural–functional studies of CHD2. Evolutionary conservation of residues 545 and 1270 was assessed using the ConSurf server. All structural images were prepared in PyMOL and UCSF ChimeraX using ray-tracing rendering (ray 1800 command in PyMOL, supersample 2 in ChimeraX).

## 6. Conclusions

In conclusion, this study presents a clinical and molecular-genetic characterization of a proband with developmental and epileptic encephalopathy resulting from the additive effect of two previously undescribed *CHD2* gene variants located in *cis* on the paternal allele. In silico analysis enabled the characterization of structural and functional alterations in the mutant protein at the tertiary level, thereby substantiating the probable disease mechanism.

This structural bioinformatic study has several inherent limitations. First, the three-dimensional modeling of the macromolecule was based on a de novo structure predicted by the AlphaFold platform, which may harbor local conformational inaccuracies. Second, the predictive algorithms employed (FoldX, mCSM, and others) represent static thermodynamic approaches that operate with approximate energy parameters and are unable to fully account for the complex kinetics of protein folding, the involvement of molecular chaperones, or the specificity of the cellular microenvironment. Finally, the proposed hypothesis of functional haploinsufficiency requires further verification using direct experimental methods in vitro (assessment of protein stability and its transcriptional activity) and in vivo.

Nevertheless, despite these limitations, the high degree of evolutionary conservation of the studied loci, the calculated destabilization values of the tertiary structure, and the phenotypic similarity of the proband to classical manifestations of *CHD2*-associated pathology allow us to consider the identified variants, both in their cumulative manifestation and individually, with a high degree of confidence as the pathogenetic cause of the disease in this patient.

## Figures and Tables

**Table 1:** Phasing results and representative markers used.

Variant	Position	Ref>Alt	Proband	Father	Mother	Interpretation
c.1634T>G (p.Leu545Arg)	92953488 (variant)	T>G	1|0	0/0 (absent)	0/0 (absent)	De novo variant—absent in both parents, allele “0” paternal
	92953644	C>T	0|1	0/0 (absent)	1/1 (marker)	Maternal haplotype marker (homozygote 1/1)
	92954892	A>G	0|1	0/0 (absent)	1/1 (marker)	Maternal haplotype marker (homozygote 1/1)
	92956155	A>G	0|1	0/1	1/1 (marker)	Maternal haplotype marker (homozygote 1/1)
c.3810G>C (p.Leu1270Phe)	92997328 (variant)	G>C	1|0	0/0 (absent)	0/0 (absent)	De novo variant—absent in both parents, allele “0” paternal
	92993436	G>A	0|1	0/1	1/1 (marker)	Maternal haplotype marker (homozygote 1/1)
	92997767	C>T	0|1	0/1	1/1 (marker)	Maternal haplotype marker (homozygote 1/1)
	92997959	A>AC	0|1	0/1	1/1 (marker)	Maternal haplotype marker (homozygote 1/1)

Note: “0” denotes the reference (Ref) allele, “1” denotes the alternative (Alt) allele. The proband’s genotype is indicated in the format “paternal allele|maternal allele.” Each genomic region contains informative markers where the mother is homozygous for the alternative allele and the father carries at least one reference allele.

**Table 2:** Thermodynamic parameters of CHD2 variants calculated by FoldX.

Model	Total Energy (kcal/mol)	ΔΔG (kcal/mol)	Interpretation
WT (Leu545/Leu1270)	1724.96	0.00	Reference
Mut1 (p.Leu545Arg)	1734.59	+9.63	Significant destabilization
Mut2 (p.Leu1270Phe)	1729.98	+5.02	Significant destabilization
Double cis (p.Leu545Arg + p.Leu1270Phe)	1740.07	+15.11	Extreme destabilization

**Table 3:** Consensus stability change assessment (ΔΔG) by independent methods.

Algorithm	p.Leu545Arg (Helicase Domain)	p.Leu1270Phe (DNA Binding Domain)
FoldX	+9.63 (destabilization)	+5.02 (destabilization)
mCSM	−1.366 (destabilization)	−1.427 (destabilization)
DynaMut2	−0.76 (destabilization)	−0.96 (destabilization)
SDM	−1.55 (destabilization)	−1.39 (destabilization)
DUET (consensus)	−1.292 (destabilization)	−1.716 (destabilization)

**Table 4:** Evolutionary conservation of positions according to ConSurf data.

Position (WT)	Conservation Score	Status	Interpretation
LEU545	8 (of 9)	b (buried)	Conserved residue, buried in the hydrophobic core
LEU1270	9 (of 9)	b s (buried structural)	Highly conserved structural residue

**Table 5:** Clinical features of the patient compared to OMIM 615369 criteria.

Clinical Features (OMIM 615369)	Index Patient
Epileptic encephalopathy	+
Delayed psychomotor development	−
Seizures (myoclonic, atonic, febrile, absence, tonic–clonic)	+
Status epilepticus	+
Psychomotor regression	+
Intellectual disability	+
Abnormal EEG	+
Autism spectrum disorder	−
Photosensitivity	+
Onset between ages 1 to 3 years	moderate
Patients can have multiple seizure types	+

**Table 6:** Primers for long-range PCR.

Genetic Variant	Primers	Sequence
chr15:92953488T>G (NM_001271.4: c.1634T>G, p.Leu545Arg)	p.Leu545Arg-forward	5′-ATTCCCCTCCCTTCACTCCA-3′
	p.Leu545Arg-reverse	5′-AAGCAAACAAAATCCCTGCGA-3′
chr15:92997328G>C (NM_001271.4: c.3810G>C, p.Leu1270Phe)	p.Leu1270Phe-forward	5′-GCCTGTTCATTCATGTCTCTCTG-3′
	p.Leu1270Phe-reverse	5′-CCTCCCCAACCACCCAAAATA-3′

## Data Availability

The original contributions presented in this study are included in the article. Further inquiries can be directed to the corresponding author.
